# Broadband Bi-Directional Polarization-Insensitive Metamaterial Absorber

**DOI:** 10.3390/ma14237339

**Published:** 2021-11-30

**Authors:** Feng Tian, Xia Ma, Han Hao, Xuewen Li, Jingdao Fan, Liang Guo, Xiaojun Huang

**Affiliations:** 1 College of Communication & Information Engineering, Xi’an University of Science and Technology, Xi’an 710054, China; tianfeng@xust.edu.cn (F.T.); 18392555014@163.com (X.M.); haohanantenna@163.com (H.H.); 2 College of Safety Science and Engineering, Xi’an University of Science and Technology, Xi’an 710054, China; lixw@xust.edu.cn (X.L.); hxj0212@126.com (J.F.); 3 College of Physics and Electrical Engineering, Kashi University, Kashi 844007, China; guol-ks@163.com

**Keywords:** broadband, bi-direction, metamaterial, absorber

## Abstract

Conventional metamaterial absorbers eliminate the transmitted electromagnetic wave by attaching the metal plate with the unidirectional absorption performance; these absorbers limit the practical applications to a large extent. In this paper, we present a broadband bi-directional metamaterial absorber by etching chip resistors on the resonators for expanding the bandwidth, and two orthogonal I-shaped structures are pasted on the both sides of the ultra-thin substrate (FR-4) instead of the metal plate for enhancing absorptance of the absorber. Simulated results show that absorptance of the designed absorber is larger than 0.9 in 1.43–2.51 GHz along the forward and backward directions under both TE and TM polarizations. Microwave experiments in the chamber are performed to verify the simulations, and the experimental results exhibit the excellent agreement with the simulations. Additionally, two I-shaped structures are orthogonally pasted on an ultrathin substrate, leading to the impedance-matching of both forward and backward directions, and the absorptance can be tailed dynamically via the middle layer of the substrate. The physics of the absorption are visualized by using a transmission line based on equivalent circuits. We claim that the designed bi-directional metamaterial absorber can be a good candidate for electromagnetic stealth and energy harvesting.

## 1. Introduction

Metamaterial perfect absorbers (MPAs) have had prominent civil and military applications in electromagnetic (EM) stealth [1,2], EM energy harvesting [3,4,5,6,7], RCS reduction [8], sensors [9,10,11,12,13], and other fields [14,15,16,17,18,19,20] since the MPA was first introduced by Landy et al. [21]. MPA-based energy harvesters can transfer the electric energy to the nodes in a wireless sensor network and eliminate the undesirable EM radiations around electronic equipment. A great deal of effort has been made on the design and implementation of multi-band [22,23,24,25] and broadband MPAs [1,2,26,27,28,29,30] via tailing the various of patterns and structures. Furthermore, MPAs with good polarization-insensitive performance and oblique incident tolerance have been realized by different methods [24,31,32,33,34]. Recently, the tunable/switchable MPAs by loading the varactor or PIN diodes have also been achieved by plentiful research achievements [35,36,37,38,39].

Most of the conventional MPAs are unidirectional absorbers because of the metal plate attached to the back for eliminating the transmitted EM waves, and the drawbacks of which limit the practical applications to a great extent. Therefore, bidirectional MPAs with good performance of polarization tolerance are highly desirable for the significant demand and potential applications. Researchers have tried to design bidirectional absorbers using various ways [40,41,42], but still suffer from the difficulties of the bandwidth, absorptance, and polarization tolerance. Few previous bidirectional absorbers have different absorption performances in different directions, and manifest the polarization sensitivity under TE and TM waves in two opposite directions. Stephen presented a bidirectional, bandwidth-enhanced metamaterial absorber with basic elements of strips and squares, which exhibited more than 0.9 absorption between 13.40 GHz and 14.25 GHz from the two incident directions; however, the fractional bandwidth was only 6.15% [40]. This limits the practical applications.

The bidirectional absorber proposed in this paper, covering the frequency range of GSM, 3G, and Wi-Fi, thus can be used in the wireless communication system. However, absorbers in those bands have been proposed as direction-sensitive in the existing literature, which limits its practical applications. In this paper, we propose a broadband bi-directional polarization-insensitive MPA working at 1.43–2.51 GHz to efficiently use electromagnetic energy in the environment. The bandwidth of the designed MPA is extended by etching the chip resistors on the unit cell resonators; and two orthogonal I-shaped resonators are pasted on both sides of the ultra-thin substrate (FR-4) to enhance the absorptance. Simulated absorptance of the designed absorber with the polarization-tolerance performance is larger than 0.9 in 1.43–2.51 GHz along the forward (+k) and backward (−k) directions. The fractional bandwidth of this work is 55%. Perfect bidirectional absorber realized in this way can broaden the bandwidth by a great deal. Microwave experiments in the chamber are performed to verify the simulations. Significantly, the ultra-thin substrate of the middle layer is of importance in the impedance-matching of both forward and backward directions, and the absorptance can be tailed dynamically via the middle layer of the substrate. In addition, the physics of the absorption is visualized by using equivalent circuits based on the transmission line theory.

## 2. Design and Simulation

**Figure 1:**
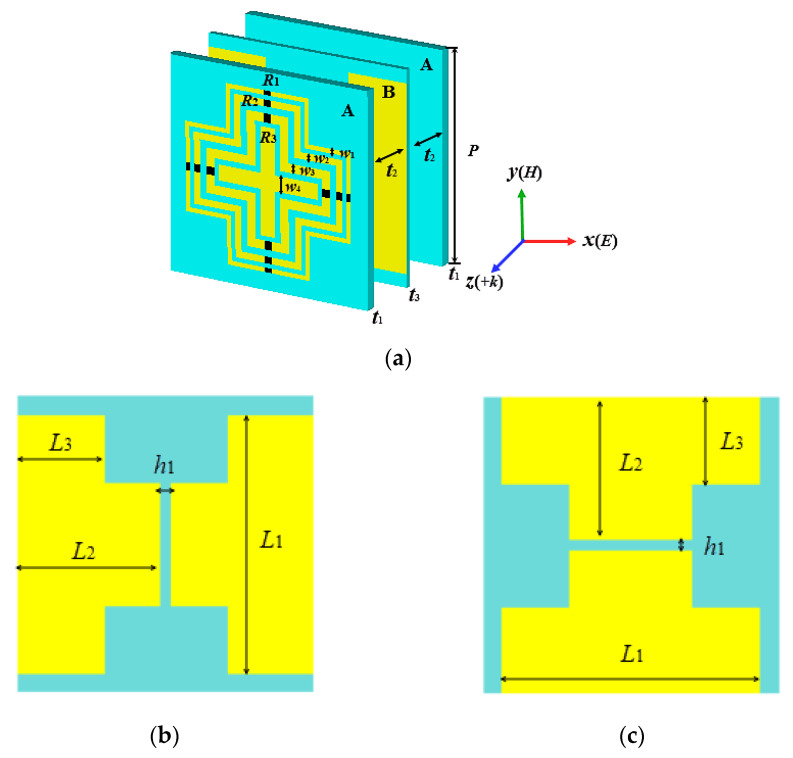
The designed absorber. (**a**) Lateral view of the absorber; (**b**) front view and (**c**) back view of layer B.

Figure 1 shows the unit cell of the designed absorber and the unit cell resembles a sandwich-shaped A-B-A geometry. Layer A, shown in Figure 1a, consists of a cross and hollow crosses, and the chip resistors are etched on the hollow crosses. Layer B, shown in Figure 1b,c, is composed of two orthogonal I-shaped structures pasted on both sides of the ultra-thin substrate. The ultra-thin substrate is of significance in the impedance-matching of both forward and backward directions. The substrates of layers A and B are FR-4 with a permittivity of 4.3 and loss tangent of 0.025. The structures of the MPA are copper, with a thickness of 0.035 mm and conductivity of 5.8 × 10^7^ S/m. The optimized parameters of the geometry are shown in Table 1.

The numerical simulation is calculated by using CST. The unit cell boundary is applied in the *x* and *y* directions to mimic infinite boundaries, and the open (add space) boundary is set in the *z* direction to represent the propagation of EM waves. When the incident wave is vertical to the upper surface of the MPA, the absorptance A is calculated from the S-parameters by A = 1 − |S_21_|^2^ − |S_11_|^2^, where S_11_ is the reflectance and S_21_ is the transmittance.

## 3. Results and Discussion

**Figure 2:**
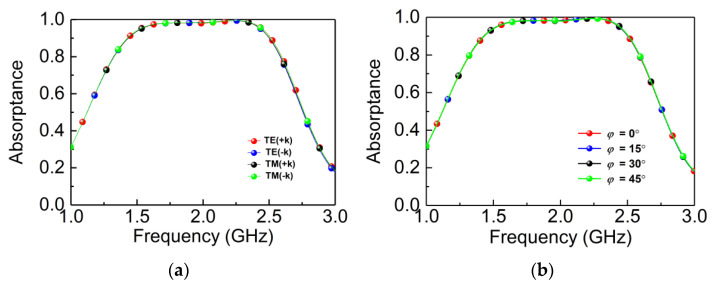
Absorptance with different polarization angles. (**a**) Forward and backward absorptance in TE and TM mode; (**b**) absorptance at different polarization angles. +k represents the EM wave along the +*z* direction, and −k represents the EM wave along −*z* direction.

Figure 2a shows the simulated absorptance along the forward and backward directions both in TE and TM modes. We find that the absorptance is greater than 0.9 in 1.43–2.51 GHz with the fractional bandwidth of 55%, and all the results coincide with each other perfectly due to the symmetric geometry of the structure, and the absorptance remains constant at all the polarization angles from 0° to 45°. Thus, the absorber has good absorption robustness of the TE and TM polarizations.

**Figure 3:**
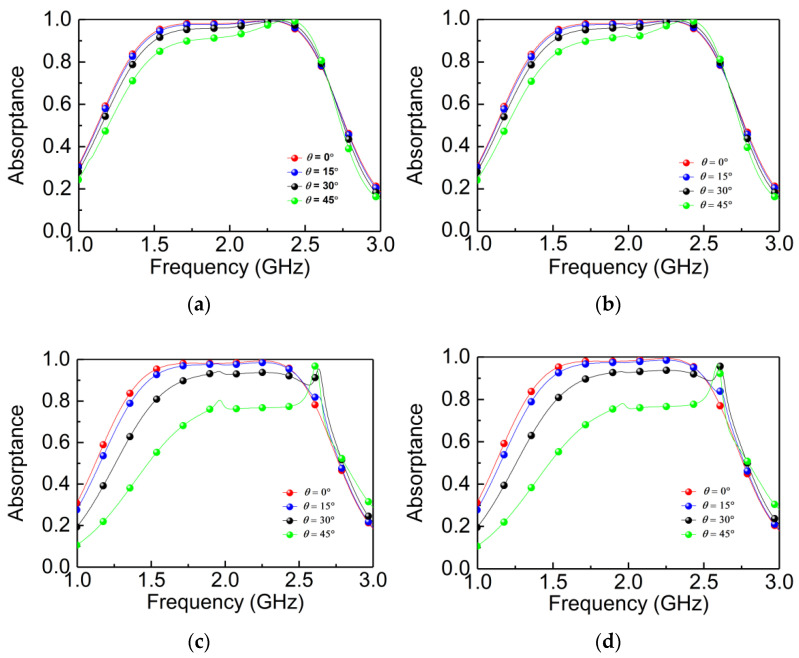
Absorptance with different oblique incidence. (**a**) Absorptance under TE wave along the −*z* direction; (**b**) absorptance under TE wave along the *+z* direction; (**c**) absorptance under TM wave along the −*z* direction; (**d**) absorptance under TM wave along the *+z* direction.

Figure 3 shows the absorptance under oblique incidence in TE and TM waves along *+z* and *−z* directions, respectively. The simulation results show that the absorptance of TE wave along −*z* and *+z* directions are both larger than 0.8 in 1.43–2.51 GHz with the incident angle reaching 45°, shown in Figure 3a,b. For the TM wave, the absorptance along the −*z* and *+z* directions also remain 0.7 at 1.43–2.51 GHz when the incident angle reaches 45°, as shown in Figure 3c,d. Specifically, we need to emphasize the difference between the EM coupling of TE and TM waves, and the absorptance of the bidirectional MPA is mainly due to magnetic coupling. The H-field direction of the TE wave is along the *y*-axis, and there is coupling in this case, so the magnetic coupling will not be weakened as the incident angle increases. For the TM wave, the H-field direction along the *x*-axis as well as the absorptance decreases gradually with the increase of the incident angle because of the decrease in magnetic coupling. However, it is satisfied that the absorptance does not decrease significantly, which also exceeds 0.7 as the incident angle increases, ranging from 0° to 45° in the case of TM waves in Figure 3c,d.

**Figure 4:**
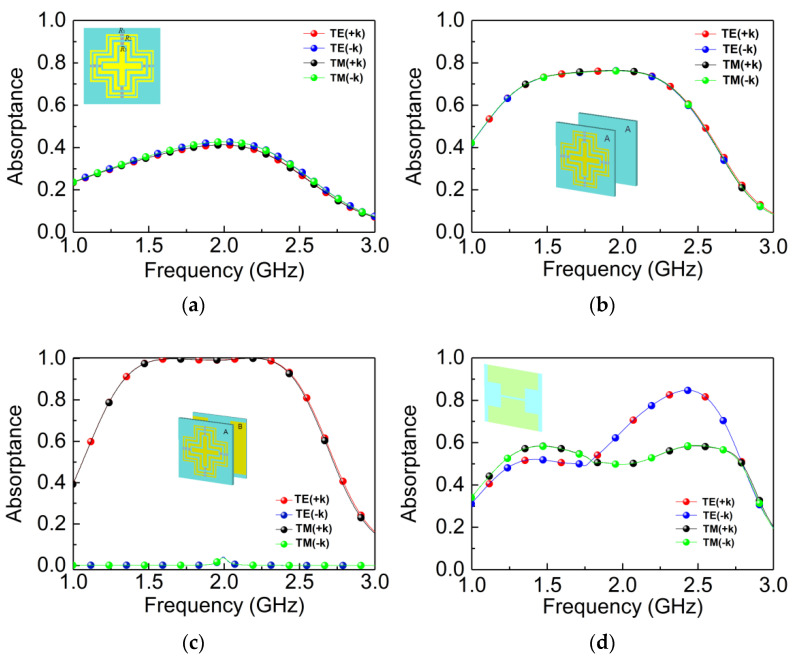
Absorptance under different combination structures. (**a**) Single A structure; (**b**) A-A structure; (**c**) A-B structure; (**d**) parallel-pasted I-shaped structures of the B layer.

Figure 4 shows the design evolution of the designed absorber. Figure 4a shows the simulated absorptance of a single A layer of the designed MPA. It can be seen from Figure 4a that the maximum absorptance is 0.4 in 2.01 GHz. It means more than 60% of the energy of the EM wave is reflected and transmitted, which ascribes to the impedance mismatching under this condition. Next, the structure of the double layers of A is simulated in Figure 4b, and the absorptance is larger than 0.75 in 1.35–2.34 GHz. In what follows, the direction-insensitive absorber is designed by introducing layer B to layer A. This procedure leads to the forward-perfect absorption, while the backward absorptance approaches zero, as shown in Figure 4c. The absorptance (nearly perfect absorption) is dramatically increased by adding layer B, and thus layer B plays an indispensable role in the perfect absorption.

In Figure 2, we have already demonstrated the perfect absorption realized by two orthogonal I-shaped structures pasted on both sides of the ultra-thin substrate. However, when two I-shaped structures are pasted in parallel, the absorptance is completely different. As showed in Figure 4d, the absorption spectrum for the TE mode appears as a peak in both forward and backward directions. Additionally, the absorptance for the TM mode is lower than 0.6 in both the forward and backward directions. Therefore, in order to realize bidirectional absorption of the given absorber, we have to break the symmetry of the B layer. In addition, the orthogonal symmetry structure can lead to the same absorption characteristics for both TE and TM waves.

**Figure 5:**
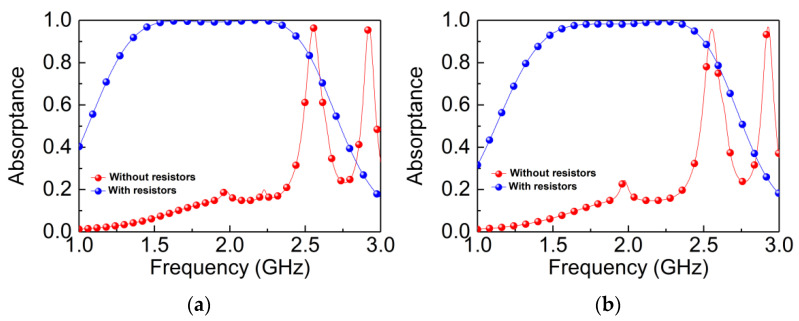
Simulated absorptance with and without resistors: (**a**) TE wave; (**b**) TM wave.

Herein, we also discuss the geometric influence of the directional insensitive MPA. To avoid the design complexity, parameters have a significant effect on absorptance, including the thickness of the air, substrate of the B layer, and the value of resistors. Figure 5a,b shows the absorptance with and without resistors on both TE and TM waves. As the designed MPA has good performances of polarization-insensitivity and wide-angle incidence, the absorptance is basically identical under both TE and TM polarization incidence with or without resistors. The absorptance is generally less than 0.2 below 2.35 GHz without resistors, and this may be due to impedance mismatches below 2.35 GHz, and two strong resonant peaks with nearly perfect absorption (A > 0.95) also appear at 2.54 and 2.93 GHz. From the electric and magnetic energy distribution (not shown in the paper), we can see that these two strong resonances are excited at the gaps of the rings, and the resonant peak at 2.54 GHz is mainly caused by the outermost hollow cross gap, as the resonance at 2.93 GHz is excited by the cross-resonator gap. When loading the resistors, it is found that the two resonant peaks disappear and form a broadband absorption (A > 0.9) in 1.43–2.51 GHz. This is mainly because the resonant structures of the two resonant places are destroyed after loading resistors, and the resistors are capable of consuming EM waves. Thus, the resistors contribute a lot to the perfect broadband absorptance.

**Figure 6:**
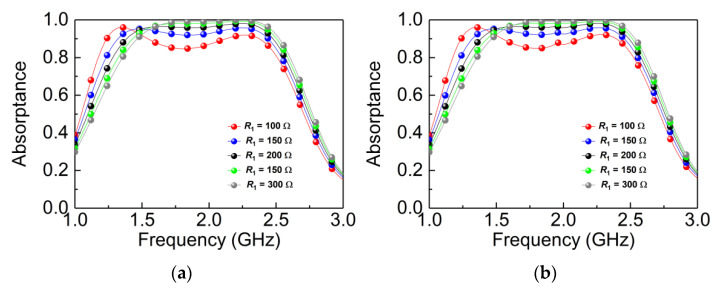

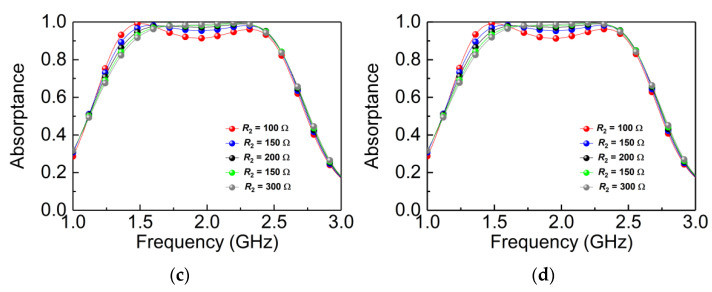
Simulated absorptance with different values of *R*_1_, *R*_2_: (**a**,**c**) TE wave; (**b**,**d**) TM wave.

Figure 6 shows the influences of the resistors of *R*_1_ and *R*_2_ under TE and TM polarization incidence, respectively. Since the designed absorber is a C4 structure, *R*_1_ and *R*_2_ have the same effect on TE and TM waves. In Figure 6a,b, it can be found that the absorptance gradually increases as the resistors of *R*_1_ increase from 100 to 300 Ω until it approaches perfect absorption. This is because the equivalent impedance of the MPA and the impedance of free space are gradually achieving a perfect match with the increase of resistors. At the same time, when the resistors of *R*_1_ increase from 100 to 300 Ω, the absorptive spectrum regularly exhibits a slight blueshift, and this phenomenon is because *R*_1_ produces a distribution effect; when *R*_1_ changes, the absorption spectrum appears to be a frequency shift. In addition, the blueshift phenomenon below 1.5 GHz is obviously better than other frequency bands, and this is because *R*_1_ are loaded in the outermost hollow cross-resonator, which mainly contributes to the absorption of low frequency. Figure 6c,d shows the influences of absorptance when adjusting the resistor of *R*_2_, while keeping *R*_1_ and *R*_3_ fixed. It can be seen that the absorptance gradually increased with the increase of *R*_2_ in 1.43–2.51 GHz, but there is no red shift phenomenon, so we conclude that *R*_2_ cancels the blueshift effect caused by *R*_1_.

**Figure 7:**
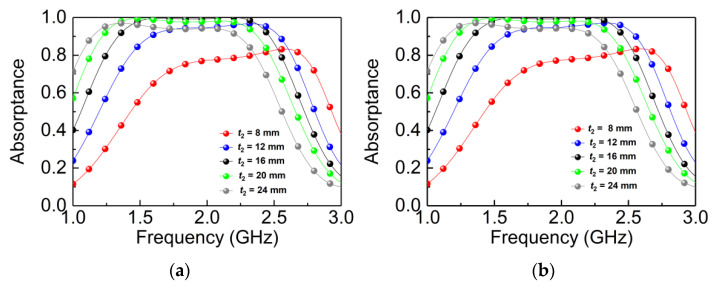
Simulated absorptdance with different values of *t*_2_: (**a**) TE wave; (**b**) TM wave.

Figure 7a,b shows the absorptance with different thicknesses of the air (*t*_2_) between layers A and B. When changing *t*_2_ from 8 mm to 24 mm, the bandwidth is narrowed, the absorptance spectrum shows a redshift, and the absorbance decreases significantly. While changing the thickness of the air, the capacitance between structures A and B changes, and the absorptance spectrum thus exhibits a redshift. Additionally, the increase of air thickness is similar to increasing the dielectric thickness, which will affect the impedance of the absorber and lead to mismatching with the free space.

**Figure 8:**
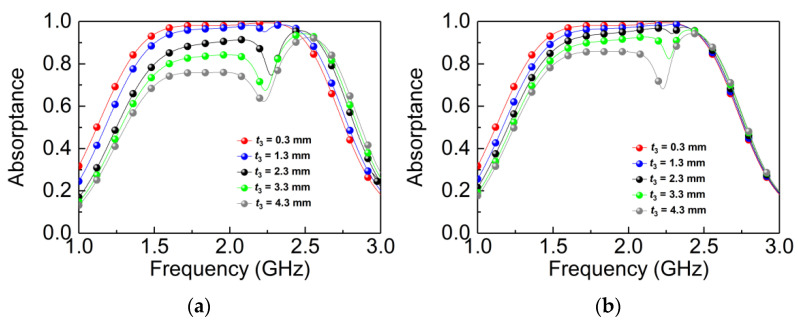
Simulated absorptance with different values of *t*_3_: (**a**) TE wave; (**b**) TM wave.

Absorption spectra of the substrate of FR-4 (*t*_3_) of the B layer with different thicknesses were simulated in TE and TM modes, as shown in Figure 8a,b, respectively. When changing the thickness of FR-4 from 0.3 mm to 4.3 mm while fixing other parameters, a distinct splitting point appears, which is caused by the slot of the I-shaped resonators. Additionally, with the increase of *t*_3_, the absorptance decreases gradually. According to the transmission line theory (discussed in more detail in part 4), as the thickness of the FR-4 increases, the equivalent impedance of the absorber will be changed, which leads to the mismatch between the impedance of the absorber and the free space. In addition, the substrate of FR-4 has different effects on the absorptance of the TE wave and TM wave. This is because the B structure is not the perfect C4 structure, which is realized by covering two orthogonal I-type resonators on the ultra-thin FR-4 dielectric, and with the increase of *t*_3_, the C4 structure will be destroyed, resulting in different absorptances of the TE wave and TM wave. The results show that the absorptance can be tailed dynamically via the middle layer of the substrate.

## 4. Analysis of the Equivalent Circuit

**Figure 9:**
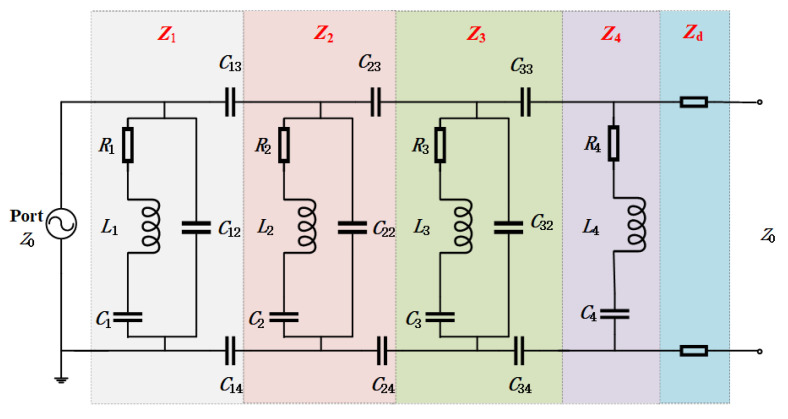
Equivalent circuit model of the proposed absorber.

The equivalent circuit layout of the investigated MPA is shown in Figure 9. In TE polarization, the metal wires are connected in the vertical direction to produce an inductance effect, and the slit produces a capacitance effect, thus forming a series resonator with the lumped resistors. The gap between the inner and outer rings of the metal in the horizontal direction produces the capacitance effect, which forms a series resonator with the inner metal ring. Due to the symmetrical property of the structure, the MPA in TM polarization has the identical equivalent circuit model. The parameters of the equivalent circuit are shown in Table 2.

The equivalent admittance of each single resonator can be obtained as follows [43,44]:

(1) $$Y_{1}=\frac{1}{R_{1}+j\omega L_{1}+\frac{1}{j\omega C_{1}}}+j\omega C_{12}+\frac{1}{j\omega C_{13}}+\frac{1}{j\omega C_{14}}$$

(2) $$Y_{2}=\frac{1}{R_{2}+j\omega L_{2}+\frac{1}{j\omega C_{2}}}+j\omega C_{22}+\frac{1}{j\omega C_{23}}+\frac{1}{j\omega C_{24}}$$

(3) $$Y_{3}=\frac{1}{R_{3}+j\omega L_{3}+\frac{1}{j\omega C_{3}}}+j\omega C_{32}+\frac{1}{j\omega C_{33}}+\frac{1}{j\omega C_{34}}$$

(4) $$Y_{4}=\frac{1}{R_{4}+j\omega L_{4}+\frac{1}{j\omega C_{4}}}$$

The equivalent admittance of the resonators can be expressed as:

(5) YRe=Y1+Y2+Y3+Y4

The equivalent impedance can be expressed as:

(6) ZRe=1YRe

According to the transmission line theory (TLM), the equivalent impedance of the dielectric substrate is:

(7) $$Z_{d}=jZ_{0}\sqrt{\frac{1}{\varepsilon_{r}}}\tan(\frac{2\pi f}{c}\sqrt{\varepsilon_{r}d})$$

Therefore, the equivalent impedance of the proposed absorber can be expressed as:

(8) Zr=ZRe×ZdZRe+Zd

**Figure 10:**
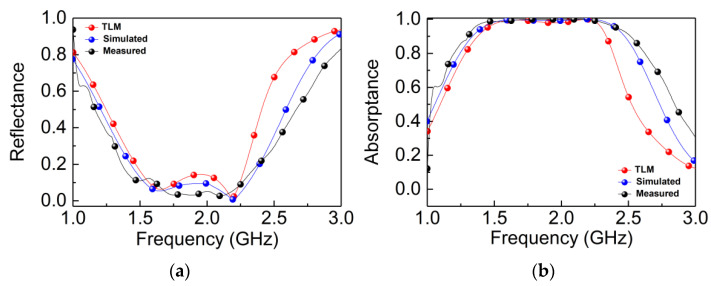
Simulated, measured, and TLM (**a**) reflectance and (**b**) absorptance of the proposed absorber.

The reflection coefficient of the absorber can be obtained as follows:

(9) $$\Gamma_{r}=20\mathrm{lg}\left|\frac{Z_{r}-Z_{0}}{Z_{r}+Z_{0}}\right|$$

where Z_0_ represents the wave impedance of free space, *ε*_r_ represents the dielectric constant of the dielectric substrate, and *d* is the thickness of the dielectric substrate. The reflectance and absorptance simulated in Advanced Design System (ADS) software according to TLM is shown in Figure 10. It is found that the reflectance and absorptance of TLM, both simulated and measured, basically coincide.

## 5. Experimental Verification

**Figure 11:**
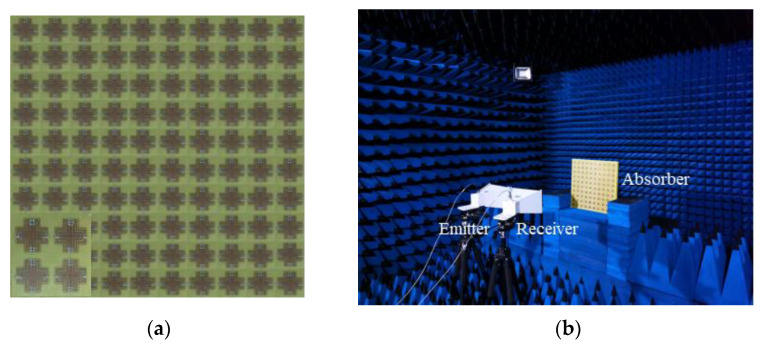
(**a**) Photograph of the fabricated sample; (**b**) photograph of the experimental environment.

For the experiment, the measured sample (50 × 50 cm^2^) with the identical geometry with the simulation was fabricated by using standard printed circuit board (PCB) technology. Four holes were drilled in four corners of the sample with a diameter of 1 cm to fix the MPA for the experimental verification, and the experimental sample is shown in Figure 11a. The reflection and transmission parameters were measured by using a pair of identical standard broadband horn antennas (1–18 GHz), which connected to the Vector Network Analyzer (Agilent E8362B) via cables in the anechoic chamber, as shown in Figure 11b. The distance between the horn antenna and MPA was maintained to avoid the near field effects. Additionally, a metal plate with the same size of the sample was used for calibration before the test.

**Figure 12:**
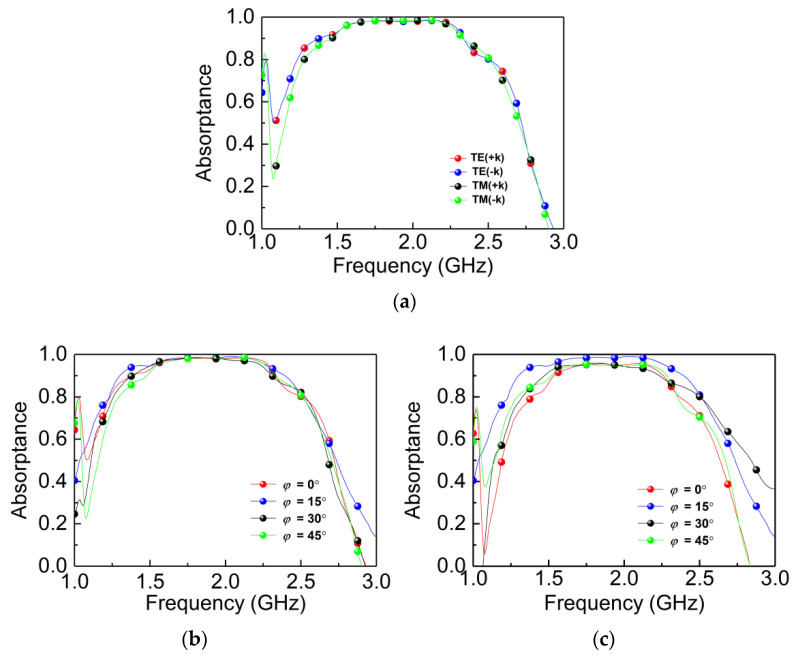
Measured absorption properties of the designed absorber. (**a**) Forward and backward absorptance in TE and TM mode; (**b**,**c**) absorptance responses of TE and TM modes vary with frequencies at different polarization angles.

The experimental results follow the same trend as the simulation shown in Figure 12. Figure 12a shows the measured absorptance for forward and backward incidence under both TE and TM polarizations, and we can see that the absorptance is greater than 0.9 in 1.43–2.51 GHz with a fractional bandwidth of 55%. Figure 12b,c shows that the bi-directional MPA is polarization-insensitive for both TE and TM polarizations. The measured results basically coincide with the simulated results. These experimental results prove that our previous simulations are basically correct.

## 6. Conclusions

In conclusion, we have numerically and experimentally proposed a broadband bi-directional polarization-insensitive MPA working at 1.43–2.51 GHz to efficiently use electromagnetic energy in the environment. The absorptance of the designed absorber is larger than 0.9 in 1.43–2.51 GHz along the forward and backward directions under both TE and TM polarizations. The absorptance was also shown to keep a high value under both TE and TM polarizations, with an incident angle up to 45°. The design evolution elaborated how the structure contributes to the direction-insensitivity absorption. What counts is that the ultra-thin substrate of the middle layer is of importance in the impedance matching of both forward and backward directions. Microwave experiments in the chamber were performed to verify the simulations, and the experimental results exhibited excellent agreement with the simulations. The transmission line theory was introduced to effectively visualize the physics of the designed absorber’s absorption. We claim that the designed bi-directional metamaterial absorber can be a good candidate for electromagnetic stealth and energy harvesting.

## Figures and Tables

**Table 1:** Optimized dimensions of the unit cell.

**Parameter**	***R*_1_**	***R*_2_**	***R*_3_**	***P***	***L*_1_**	***L*_2_**	***L*_3_**	***h*_1_**
Dimension	250 Ω	250 Ω	450 Ω	48 mm	42 mm	23.15 mm	14 mm	1.7 mm
**Parameter**	***t*_1_**	***t*_2_**	***t*_3_**	***w*_1_**	***w*_2_**	***w*_3_**	***w*_4_**	**--**
Dimension	2.5 mm	14 mm	0.3 mm	1 mm	1.5 mm	2 mm	3.5 mm	--

**Table 2:** Optimized parameter value of the equivalent circuit model.

**Parameter**	***R*_1_**	***R*_2_**	***R*_3_**	***R*_4_**	**Parameter**	***C*_2_**	***C*_22_**	***C*_23_**	***C*_24_**
Value (Ω)	30.01	0.01	900.01	0.01	Value (pF)	2.75	0.66	17.12	0.61
**Parameter**	***L*_1_**	***L*_2_**	***L*_3_**	***L*_4_**	**Parameter**	***C*_3_**	***C*_32_**	***C*_33_**	***C*_34_**
Value (nH)	27.31	4.96	0.01	24.01	Value (pF)	1.21	0.01	11.11	0.88
**Parameter**	***C*_1_**	***C*_12_**	***C*_13_**	***C*_14_**	**Parameter**	***C*_4_**	**--**	**--**	**--**
Value (pF)	0.31	0.28	0.01	1.55	Value (pF)	3.61	--	--	--

## Data Availability

The data presented in this study are available on request from the corresponding author.

## References

[1] Kim J., Han K., Hahn J.W. (2017). Selective dual-band metamaterial perfect absorber for infrared stealth technology. Sci. Rep..

[2] Peng L., Liu D., Cheng H., Zhou S., Zu M. (2018). A multilayer film based selective thermal emitter for infrared stealth technology. Adv. Opt. Mater..

[3] Li P., Liu B., Ni Y., Liew K.K., Sze J., Chen S. (2015). Large-scale nanophotonic solar selective absorbers for high-efficiency solar thermal energy conversion. Adv. Mater..

[4] Lin K.-T., Lin H., Yang T., Jia B. (2020). Structured graphene metamaterial selective absorbers for high efficiency and omnidirectional solar thermal energy conversion. Nat. Commun..

[5] Alavikia B., Almoneef T.S., Ramahi O.M. (2015). Wideband resonator arrays for electromagnetic energy harvesting and wireless power transfer. Appl. Phys. Lett..

[6] Duan X., Chen X., Zhou Y., Zhou L., Hao S. (2018). Wideband metamaterial electromagnetic energy harvester with high capture efficiency and wide incident angle. IEEE Antennas Wirel. Propag. Lett..

[7] Alkurt F.O., Altintas O., Ozakturk M., Karaaslan M., Akgol O., Unal E., Sabah C. (2020). Enhancement of image quality by using metamaterial inspired energy harvester. Phys. Lett. A.

[8] Liu T., Cao X., Gao J., Zheng Q., Li W., Yang H. (2012). RCS Reduction of Waveguide Slot Antenna with Metamaterial Absorber. IEEE Trans. Antennas Propag..

[9] Bhattarai K., Ku Z., Silva S., Jeon J., Kim J.O., Lee S.J., Urbas A., Zhou J. (2015). A large-area, mushroom-capped plasmonic perfect absorber: Refractive index sensing and Fabry—Perot cavity mechanism. Adv. Opt. Mater..

[10] Cetin A.E., Korkmaz S., Durmaz H., Aslan E., Kaya S., Paiella R., Turkmen M. (2016). Quantification of multiple molecular fingerprints by dual-resonant perfect absorber. Adv. Opt. Mater..

[11] Vafapour Z. (2019). Polarization-independent perfect optical metamaterial absorber as a glucose sensor in food industry applications. IEEE Trans. Nanobiosci..

[12] Hasan D., Lee C. (2018). Hybrid metamaterial absorber platform for sensing of CO2 gas at Mid-IR. Adv. Sci..

[13] Lari E.S., Vafapour Z., Ghahraloud H. (2020). Optically tunable triple—band perfect absorber for nonlinear optical liquids sensing. IEEE Sens. J..

[14] Chen K., Adato R., Altug H. (2012). Dual-band perfect absorber for multispectral plasmon-enhanced infrared spectroscopy. ACS Nano.

[15] Zhao L., Liu H., He Z., Dong S. (2018). All-metal frequency-selective absorber/emitter for laser stealth and infrared stealth. Appl. Opt..

[16] Chirumamilla M., Chirumamilla A., Yang Y., Roberts A.S., Kristensen P.K., Chaudhuri K., Boltasseva A., Sutherland D.S., Bozhevolnyi S.I., Pedersen K. (2017). Large-area ultrabroadband absorber for solar thermophotovoltaics based on 3D titanium nitride nanopillars. Adv. Opt. Mater..

[17] Kim I., So S., Rana A.S., Mehmood M.Q., Rho J. (2018). Thermally robust ring-shaped chromium perfect absorber of visible light. Nanophotonics.

[18] Zuo W., Yang Y., He X., Zhan D., Zhang Q. (2017). A miniaturized metamaterial absorber for ultrahigh—frequency RFID system. IEEE Antennas Wirel. Propag. Lett..

[19] Li A., Singh S., Sievenpiper D. (2018). Metasurfaces and their applications. Nanophotonics.

[20] Bilal R.M.H., Baqir M.A., Choudhury P.K., Ali M.M., Rahim A.A., Kamal W. (2020). Polarization-insensitive multi-band metamaterial absorber operating in the 5G spectrum. Opt.-Int. J. Light Electron Opt..

[21] Landy N.I., Sajuyigbe S., Mock J.J., Smith D.R., Padilla W.J. (2008). Perfect metamaterial absorber. Phys. Rev. Lett..

[22] Wen D., Huang X., Guo L., Yang H., Han S., Zhang J. (2015). Quadruple-band polarization-insensitive wide-angle metamaterial absorber based on multi-layer structure. Optik.

[23] Zhu W., Rukhlenko I.D., Xiao F., He C., Geng J., Liang X., Premaratne M., Jin R. (2017). Multiband coherent perfect absorption in a water-based metasurface. Opt. Express.

[24] Cheng Y.Z., Huang M.L., Chen H.R., Guo Z.Z., Mao X.S., Gong R.Z. (2017). Ultrathin six-band polarization-insensitive perfect metamaterial absorber based on a cross-cave patch resonator for terahertz waves. Materials.

[25] Wang L., Huang X., Li M., Dong J. (2019). Chirality selective metamaterial absorber with dual bands. Opt. Express.

[26] Lin H., Sturmberg B.C.P., Lin K.-T., Yang Y., Zheng X., Chong T.K., Sterke C.M.S., Jia B. (2019). A 90-nm-thick graphene metamaterial for strong and extremely broadband absorption of unpolarized light. Nat. Photonics.

[27] Yu P., Besteiro L.V., Huang Y., Wu J., Fu L., Tan H.H., Jagadish C., Wiederrecht G.P., Govorov A.O., Wang Z. (2019). Broadband Metamaterial Absorbers. Adv. Opt. Mater..

[28] Zhuang H.Y., Wang X.K., Wang J.J., Guo M.C., Tang D.M., Zhang B.S., Chen X., Chen P., Yang Y. (2020). Broadband microwave metamaterial absorber based on magnetic periodic elements. J. Phys. D Appl. Phys..

[29] Deng G.S., Lv K., Sun H.X., Yang J., Yin Z.P., Chi B.H., Li X. (2021). An ultra-broadband and optically transparent metamaterial absorber based on multilayer indium-tin-oxide structure. J. Phys. D Appl. Phys..

[30] Fan S., Song Y. (2020). Ultra-wideband flexible absorber in microwave frequency band. Materials.

[31] Wang B.X., Wang L.L., Wang G.Z., Huang W.Q., Li X.F., Zhai X. (2014). Theoretical investigation of broadband and wide-angle terahertz metamaterial absorber. IEEE Photonics Technol. Lett..

[32] Deng G., Lv K., Sun H., Yang J., Yin Z., Li Y., Chi B., Li X. (2020). An ultrathin, triple—band metamaterial absorber with wide - incident - angle stability for conformal applications at X and Ku frequency band. Nanoscale Res. Lett..

[33] Ghosh S., Bhattacharyya S., Kaiprath Y., Vaibhav Srivastava K. (2014). Bandwidth-enhanced polarization-insensitive microwave metamaterial absorber and its equivalent circuit model. J. Appl. Phys..

[34] Amiri M., Tofigh F., Shariati N., Lipman J., Abolhasan M. (2020). Wide—angle metamaterial absorber with highly insensitive absorption for TE and TM modes. Sci. Rep..

[35] Li A., Kim S., Luo Y., Li Y., Long J., Sievenpiper D.F. (2017). High-power transistor-based tunable and switchable metasurface absorber. IEEE Trans. Microw. Theory Tech..

[36] Li Y., Lin J., Guo H., Sun W., Xiao S., Zhou L. (2020). A tunable metasurface with switchable functionalities: From perfect transparency to perfect absorption. Adv. Opt. Mater..

[37] Jeong H., Le D.H., Lim D., Phon R., Lim S. (2020). Reconfigurable metasurfaces for frequency selective absorption. Adv. Opt. Mater..

[38] Nemati A., Wang Q., Hong M., Teng J. (2018). Tunable and reconfigurable metasurfaces and metadevices. Opto-Electron. Adv..

[39] Han C., Zhong R., Liang Z., Yang L., Fang Z., Wang Y., Ma A., Wu Z., Hu M., Liu D. (2021). Independently tunable multipurpose absorber with single layer of metal-graphene metamaterials. Materials.

[40] Stephen L., Yogesh N., Subramanian V. (2019). Realization of bidirectional, bandwidth-enhanced metamaterial absorber for microwave applications. Sci. Rep..

[41] Li Z., Liu W., Tang C., Cheng H., Li Z., Zhang Y., Li J., Chen S., Tian J. (2020). A bilayer plasmonic metasurface for polarization-insensitive bidirectional perfect absorption. Adv. Theory Simul..

[42] Meng H., Shang X., Xue X., Tang K., Xia S., Zhai X., Liu Z., Chen J., Li H., Wang L. (2019). Bidirectional and dynamically tunable THz absorber with Dirac semimetal. Opt. Express.

[43] Langley R.J., Parker E.A. (1982). Equivalent circuit model for arrays of square loops. Electron. Lett..

[44] Chen X., Li Y., Fu Y., Yuan N. (2012). Design and analysis of lumped resistor loaded metamaterial absorber with transmission band. Opt. Express.

